# Identification of the underlying factor structure of the Derriford Appearance Scale 24

**DOI:** 10.7717/peerj.1070

**Published:** 2015-07-02

**Authors:** Timothy P. Moss, Victoria Lawson, Paul White, The Appearance Research Collaboration

**Affiliations:** 1 Centre for Appearance Research, University of the West of England, Bristol, QY, UK; 2 Southwark Psychological Therapies Service, Maudsley Hospital, London, UK

**Keywords:** Self-consciousness, Sexual self-consciousness, Body, Appearance, DAS24, Derriford, Visible difference

## Abstract

**Background.** The Derriford Appearance Scale24 (DAS24) is a widely used measure of distress and dysfunction in relation to self-consciousness of appearance. It has been used in clinical and research settings, and translated into numerous European and Asian languages. Hitherto, no study has conducted an analysis to determine the underlying factor structure of the scale.

**Methods.** A large (*n* = 1,265) sample of community and hospital patients with a visible difference were recruited face to face or by post, and completed the DAS24.

**Results.** A two factor solution was generated. An evaluation of the congruence of the factor solutions on each of the the hospital and the community samples using Tucker’s Coefficient of Congruence (*r_c_* = .979) and confirmatory factor analysis, which demonstrated a consistent factor structure. A main factor, general self consciousness (GSC), was represented by 18 items. Six items comprised a second factor, sexual and body self-consciousness (SBSC). The SBSC scale demonstrated greater sensitivity and specificity in identifying distress for sexually significant areas of the body.

**Discussion.** The factor structure of the DAS24 facilitates a more nuanced interpretation of scores using this scale. Two conceptually and statistically coherent sub-scales were identified. The SBSC sub-scale offers a means of identifying distress and dysfunction around sexually significant areas of the body not previously possible with this scale.

## Introduction

The subjective experience of living with a different appearance has been well reported. Visibly different people are subject to staring and unwelcome attention, avoidance, through to overt teasing, hostility and rejection (e.g., MacGregor et al., 1953; Lansdown et al., 1997; Bundy, 2012). The resultant psychological distress and dysfunction associated with visible differences associated with disease, traumatic injury and congenital and developmental abnormality has similarly been increasingly documented over recent years (Bessell et al., 2012b). Difficulties reported include social avoidance, fear of negative evaluation, shame, and anxiety (Rosser, Moss & Rumsey, 2010). Applied psychologists, including health, clinical, and counseling psychologists have been at the forefront of developing interventions to support people with psychological needs arising from visible differences (Bessell et al., 2012a; Bessell et al., 2012b), and in developing a clearer understanding of the differentiating factors and processes between those who adjust well, and those who struggle to cope and manage with differing appearances. It is clear that the objective severity of appearance differences has little impact on the psychological adjustment to visible differences (Moss, 2005). The underlying processes which do mediate and moderate individual differences in adjustment include the perception and use of social support, the availability of negative self views within the self-concept, and attentional biases towards appearance related information in the external environment (cf. Moss & Rosser, 2012). Within the literature on body image in the wider population, the tripartite influence model of adjustment highlights the social impacts of peers, family and the media on body image, including the internalization of social ideals about appearance, and engagement in social comparison processes (Thompson et al., 1999). An extensive literature exists based on these predictors, the relationships between them and identifying factors which intervene between these predictors, psychosocial outcomes, and appearance related behaviours.

In order to be able to have a relevant, specific and well defined outcome variable to further assess these theoretical explorations, and also to make a meaningful assessment of interventions, a team of plastic surgeons and psychologists created the Derriford Appearance Scale 59 (Carr, Harris & James, 2000). In appearance psychology and body image research, outcomes which are used are often either (1) standardized, non-appearance specific measures of anxiety, depression, or self-esteem, (2) measures of appearance (dis)satisfaction which do not incorporate issues which arise from living with a visible difference, or (3) condition specific (Thompson, 2004). The Derriford Appearance Scale was appearance specific, based directly on issues identified by those with visible differences, and applicable across diverse populations. This psychometrically sound measure derived from patient reports in plastic surgery, has shown to be valid and reliable in clinical and general population samples. It has been translated into multiple languages; for example, Japanese and Nepalese (Singh et al., 2013; Nozawa et al., 2008). However, for routine use, the DAS59 is somewhat cumbersome. Carr, Moss & Harris (2005) published a shorter form of the scale, the Derriford Appearance Scale 24 (DAS24), which retained the psychometric properties of the DAS59 but was quicker for participants to complete and had greater face validity. Originally envisaged as unifactorial, the subsequent widespread use of DAS24 in medical, and psychological practice, as well as in psychological research has led to a reconsideration of the constructs DAS24 identifies, specifically if it is a multifactorial measure. Therefore, the purpose of the current study was to investigate the factor structure of DAS24 for people who have visibly different appearance.

## Method

### Ethics

The research was approved by National Research Ethics Service UK Research Ethics Committee (Central and South Bristol-05/Q2006/19), and is consistent with the Declaration of Helsinki ethical principles. Participants were recruited in accordance with ethical guidance for obtaining informed consent, which included a two-week period to consider opting into the study.

### Participants

Sample size was based on recommendations by Comrey and Lee on minimum sample size in factor analysis (Comrey & Lee, 1992). They indicate that more than 500 is very good, whilst 1,000 or more observations is excellent. For the current study, increasing sample sizes beyond 1,000 served to enhance power and provided the opportunity to obtain a wide sample over multiple clinical groupings.

Participants aged over 18 years old who self-identified as being visibly different and with fluency in written and spoken English were recruited from community and clinical settings. Six hundred and fourteen community participants were recruited through advertisements and general practice doctors’ surgeries, whilst 651 clinical participants were recruited via outpatient clinics (prosthetics, dermatology, ophthalmology and general plastics (plastics & burns), ear, nose and throat clinics (including cleft lip and palate) cancer clinics (head and neck, skin) and laser treatment. Participants were recruited from locations across the United Kingdom (Bristol, London, Bradford, Sheffield and Warwick). In total, 1,265 participants were recruited. Eight hundred and sixty seven of the whole sample were female (68.5%), 354 male (28.0%). Four hundred and seventy four of those in the community sample were female (77.2%), 120 were male (19.5%). Similarly, 393 of those in the clinic sample were female (60.4%), 234 were male (35.9%). The mean age of the whole sample was 47.3 years (range 18–91, SD 16.7 years) with the mean age in the community sample 44.9 years (range 18–91; SD 16.2 years), marginally lower than in the clinic mean age 49.7 years (range 18–89; SD 16.9 years). 783 (61.9%) of the whole sample reported being married or living with partner, 183 (14.6%) living with friends or relatives and 287 (22.9%) living alone. 81% of the whole sample were white, with the other 12% either Pakistani, Indian, Black Caribbean, Black African or other, 7% did not state their ethnicity. The percentages are similar in both the clinic and community sample.

DAS24 was included as part of a wider Appearance Research Collaboration study that was assessing adjustment to visible difference (Clarke et al., 2013). Those who agreed to participate were given a questionnaire booklet to complete at their next outpatient appointment or mailed the booklet by post. Participants self-reported demographic information, and the aspect of their physical appearance they were most sensitive about.

### Materials

DAS24 is a 24 item self-report scale measuring social anxiety and avoidance in relation to self-consciousness of appearance. Total scores range from 11 to 96 with lower scores representing lower levels of social anxiety and social avoidance. The authors reported high internal consistency, with Cronbach’s alpha coefficients *α* = .92 and six-month test-retest reliability of *r* = .68. It has also shown good convergent and discriminant construct validity with measures of social anxiety, shame, and depression, and divergent construct validity with hysteria. It includes several reverse scored items (corrected prior to analysis, and in the associated data set presented with this paper). For a detailed description of the psychometric validation of DAS24 please refer to the original article (Carr, Moss & Harris, 2005).

### Data analytic strategy

Exploratory Factor Analysis (EFA) was performed using principal axis factoring (PAF) with an oblimin oblique rotation. Tinsley & Tinsley (1987) regard PAF as the preferred extraction procedure for factor analysis as it generates reliable solutions even when communalities are low and is robust to deviations from normality (Kahn, 2006). The number of factors to retain was determined using parallel analysis and Velicer’s MAP (see Zwick & Velicer, 1986). This process was applied to the clinical sample, the community sample, and all available data.

The stability of the factor structure was assessed by comparing solutions from the clinical and non-clinical samples, testing first the similarity of Cronbach’s alpha from each sample. This was followed by application of the Larntz-Perlman procedure for testing equality of correlation matrices (see Larntz & Perlman, 1988, or Koziol et al., 1997) which was used to compare the inter-item correlation matrix structure between the clinical population and the non-clinical population, and which tests whether the underlying factor structure is consistent across clinical and non-clinical populations (see Green, 1992). Tucker’s Coefficient of Congruence was used to quantify the degree of similarity between derived factor solutions from each of the samples. This is approach quantifies the degree of similarity of factor solutions as opposed to testing for equality. The approach is applicable without the requirement to specify a prior reasoned model and avoids the risk of failing to identify goodness of fit, associated with an independent cluster model confirmatory factor analysis (ICM-CFA) with large samples.

ICM-CFA was also used to examine further the derived model. An ICM-CFA model constrains all items to have zero factor loadings on all factors other than the one they measure which is in contrast to EFA where all cross-loadings are freely estimated in EFA. Van Prooijen & Van Der Kloot (2001) consider the procedure of CFA following EFA on the same data and confirm that the ICM-CFA approach is a stringent but worthwhile test as “*if CFA cannot confirm results of EFA on the same data, one cannot expect that CFA will confirm results of EFA in a different sample or population*.” Of course, when CFA can confirm results of EFA on the same data, one can only expect, but cannot make sure, that CFA will confirm results of EFA in a different sample or population. For these reason, random samples were also used to perform EFA and then to test the CFA using unseen data. Note that the CFA was undertaken using (a) all data, (b) the clinical subsample, (c) the community sample, and (d) following Gerbing & Hamilton (1996) random samples of size *n* = 500 were used to perform EFA and with the remainder to test the CFA model. In all cases, the same ICM-CFA model was tested. The data from participants was randomly split into two mutually exclusive subsamples. One subsample (*n* = 504) was used for EFA, and the other subsample was used for CFA (*n* = 509) for the discovered model. Throughout, standard measures of fit in CFA, including the root mean squared error approximation (RMSEA), minimum discrepancy per degree of freedom (CMIN/DF), goodness-of-fit index (GFI), adjusted goodness-of-fit index (AGFI), normed fit index (NFI), non-normed fit index (NNFI), and comparative fit index (CFI). In general threshold values of less than 0.01, 0.05, 0.08 for RMSEA are indicative of excellent, good and mediocre fit (see MacCallum, Browne & Sugawara, 1996) and with RMSEA > 0.1 indicating a poorly specified model. CMIN/DF < 3 indicates an acceptable fit between hypothetical model and sample data (Kline, 1998) and CMIN/DF <5 indicating a reasonable fit (Marsh & Hocevar, 1985). GFI, AGFI, NFI, CFI and NNFI > 0.9 indicate good levels of fit between data and model with more liberal criteria of 0.85 < GFI, NFI < 0.9 and 0.8 < AGFI < 0.9 indicating an acceptable model (see for instance, Bentler, 1990; Cole, 1987; Marsh, Balla & McDonald, 1988). We further considered whether the factor structure is metric invariant for the community and clinical samples. Cheung & Rensvold (2002) recommend the change in CFI (delta-CFI) to examine lack of invariance as delta-CFI is not overly sensitive to sample size, and delta-CFI is strongly correlated with other changes in fit indices. They further recommend that delta-CFI < .01 is compatible with invariance.

Analyses of variance (ANOVAs) were then conducted on the resultant factors to identify variability by gender, recruitment method and location of participants’ areas of visible difference sensitivity.

## Results and Discussion

### Results

Any participant for whom more than 50% of the items were missing in any scale, or those who had completed less than 50% of the total scale package, were excluded. In practice this meant excluding nine from the community sample. Thus, the missing data is only a small fractional part of the database and missing values may be considered to be missing completely at random.

Firstly, data were checked for influential observations; we measured changes in the ellipsoid volume of the dataset if an observation was deleted (Chatterjee, Jamieson & Wiseman, 1991). As there were only a small number of influential observations this was acceptable. The data was also assessed to establish if the correlation between variables was high enough for meaningful extraction, which was found to be the case with Kaiser-Meyer-Olkin measure = 0.952 (KMO > .09 is generally confirmed as “marvelous”(Kaiser & Rice, 1974).

### Factor structure across the two samples

Cronbach’s alpha for the sample data is 0.929. This value is closely replicated in the data for the clinical sample (alpha = 0.927) and the non-clinical sample (alpha = 0.930). Analysis using Feldt’s test (see Feldt, 1969; Feldt, Woodruff & Salih, 1987) indicates that Cronbach’s alpha does not significantly differ between the clinical and non-clinical sample (*p* = 0.598). All 24 corrected item total correlations fall in the desirable range of between 0.30 and 0.70 (see Cano et al., 2004), and this is replicated in both the clinical population and the non-clinical population. Similarly, all inter item correlation coefficients for items on the DAS24 are positive and less than .7 which is in keeping with Cano’s criteria for item retention (see Cano et al., 2004). This is similarly replicated in the inter item correlations for both the clinical and non-clinical populations. The Larntz-Perlman procedure for testing equality of correlation matrices (see Larntz & Perlman, 1988, or Koziol et al., 1997) was used to compare the inter-item correlation matrix structure between the clinical population and the non-clinical population. This test failed to achieve statistical significance (*p* = .267) indicating that the underlying factor structure is consistent across clinical and non-clinical populations (see Green, 1992). A principal axis factor analysis indicates a two factor solution (first eigenvalue 9.723, 95% CI [9.17–10.27]; second eigenvalue 1.858, 95% CI [1.77–1.98] with all subsequent eigenvalues less than unity). A similar analysis using the data from the non-clinical sample equally gave a two factor solution (first eigenvalue 9.807, 95% CI [9.02–10.59]; second eigenvalue 1.873, 95% CI [1.72–2.02]) as did the analysis in the clinical sample (first eigenvalue 9.587, 95% CI [8.82–10.35]; second eigenvalue 1.910, 95% CI [1.76–2.06]). The difference in the first and second eigenvalue between the clinical and non-clinical samples is not statistically significant (*p* = .583, *p* = .635 respectively). The two factor solution in the clinical sample displays a high degree of congruence with the two component solution in the non-clinical sample (Tucker’s Coefficient of Congruence = .979). Lorenzo-Seva & ten Berge (2006) suggested a value for Tucker’s Coefficient of Congruence “in the range .85–.94 corresponds to a fair similarity, while a value higher than .95 implies that the two factors or components compared can be considered equal.” One this basis a two factor solution using all the available data has been derived. This two factor solution, reported in Table 1, results from an oblique rotation (direct oblimin) as there is no a priori reasoning to impose orthogonality on the solution. CFA measures of fit were essentially constant for models using (a) all data, (b) clinical (c) community sub-samples, and (d) randomly selected sample of size *n* = 500. Near identical solutions were obtained in all cases. Median values of RMSEA = 0.06, GFI = 0.86, AGFI = 0.83, NFI = 0.84, NNFI = 0.86, CFI = 0.88, and CMIN/DF = 3.84, were obtained indicating overall acceptability but without being an excellent fit. In the metric invariance assessment comparing the Community sample with the Clinic sample delta-CFI was found to be 0.004 less than the .01 threshold suggested by Cheung & Rensvold (2002). Other measures of change in fit similarly showed small values (delta-RMSEA = 0.005; delta-GFI = 0.005, delta-AGFI = 0.002, delta-NFI = 0.006, delta-CMIN/DF = 0.054). In addition, we randomly split the data into two parts. One part was used to perform EFA (*n* = 504) and the other part was used to test the ICM-CFA model (*n* = 509). Diagnostics, for this random split for model discovery and model test were extremely similar to those reported above (RMSEA = 0.07, GFI = 0.85, AGFI = 0.83, NFI = 0.83, NNFI = 0.85, CFI = 0.87, and CMIN/DF = 3.88).

**Table 1:** EFA loadings from a principal axis decomposition with an oblique rotation (direct oblimin). Loadings less than 0.4 suppressed for clarity.

Item summary	F1 GSC loading	F2 SBSC loading
Close into shell	.765	
Avoid leaving house	.755	
Feel rejected	.746	
Feel hurt	.729	
Feel confident	.694	
Feel irritable	.675	
Feel normal	.660	
Self-conscious & irritable at home	.656	
Avoid pubs/restaurants	.651	
Distressed at reflection	.603	
Distressed at social events	.592	
Feel misjudged	.558	
Feel self-conscious of feature	.530	
Adopt concealing gestures	.515	
Distressed supermarkets/dept stores	.500	
Self-conscious adverse work impact	.495	
Feel masculine/feminine	.489	
Distressed at others’ remarks	.485	
Distressed at beach		.754
Distressed at clothing limitations		.659
Avoid communal changing		.637
Avoid undressing with partner		.559
Distressed at sports/games		.520
Adverse effect on sex life		.458

Cronbach’s alpha for the GSC subscale is 0.918; alpha for the GSC subscale in the non-clinic sample (0.930) and in the clinical sample (0.926) are not significantly different (*p* = .496). Similarly, Cronbach’s alpha for the SBSC subscale is 0.803; alpha for the SBSC subscale in the non-clinic sample (0.789) and the clinic sample (0.811) do not significantly differ (*p* = .178).

### Variability in factor response by gender and recruitment method

As would be expected, men scored lower than women on both factors (i.e., were less distressed). For GSC, men’s mean = 29.6, sd = 11.5, whereas for women mean = 35.0, sd = 11.9. For SBSC, men’s mean = 6.9, sd = 4.7, whereas women’s mean = 10.5, sd = 5.6. This was significant in both cases. For GSC, *F*(1, 1, 000) = 43.389, *p* < .0001, *η*^2^ = .042, and for SBSC, *F*(1, 1,161) = 101.576, *p* < .0001, *η*^2^ = .080. In addition, the effect sizes indicate that this variation was small for GSC but medium to large for SBSC. There were also significant difference between community and clinical samples, with higher scores noted for the clinical samples; for GSC this was *F*(1, 1,035) = 9.812, *p* < .002, *η*^2^ = .009, whilst for SBSC *F*(1, 1,203) = 16.357, *p* < .0001, *η*^2^ = .013; however, the effect sizes were very small.

### Variability in factor response by area of sensitivity

For areas of the body where participants identified their main area of sensitivity in a less sexually significant location (nose or hands) GSC was significantly greater compared to those not self conscious of these body areas. This was not the case for SBSC. Furthermore, scores for participants who identified their main area of sensitivity about their appearance as a more sexually significant or concealed location of their body were significant on both GSC and SBSC, with larger effect for SBSC sizes, as indicated in Tables 2 and 3.

**Table 2:** GSC: those concerned vs those not concerned about specific body parts.

	*Df*	*F*	*η* ^2^	*P*
Nose	1, 1,035	26.835	.025	<.0001
Hands	1, 1,035	11.238	.011	<.0001
Breasts	1, 1,035	78.251	.071	<.0001
Mouth	1, 1,035	18.805	.018	<.0001
Abdomen	1, 1,035	57.290	.0052	<.0001

**Table 3:** SBSC: those concerned vs those not concerned about specific body parts.

	*Df*	*F*	*η* ^2^	*p*
Nose	1, 1,203	.788	.001	.181
Hands	1, 1,203	.356	.000	.551
Breasts	1, 1,203	154.488	.114	<.0001
Mouth	1, 1,203	6.956	.006	<.0001
Abdomen	1, 1,203	124.212	.094	<.0001

As shown in Table 2, GSC was significant, regardless of location of appearance sensitivity, with small to medium effect sizes. For SBSC, there were large effects if the area of sensitivity was the breasts or abdomen. However, if the area of sensitivity was the mouth, the results were significant but with a much smaller effect size that GSC. There was no significant difference in SBSC between those concerned about their nose (compared to those not concerned about their nose) or hands (compared to those not concerned about their hands).

### Discussion

Principal axis factoring of DAS24 generated two factors, General Self-Consciousness of appearance (GSC) and Sexual and Bodily Self-Consciousnesses of appearance (SBSC).

Women scored more highly (indicated more distress) than men on both factors. This is consistent with prior evidence and theory about gender and appearance. For example, Cash, Ancis & Strachan (1997) have demonstrated that women are the subject of greater and pervasive body scrutiny than men, and are both more dissatisfied and invested in their appearance. Forbes et al. (2007) have argued that women’s appearance fulfils a social function—to “*dissipate their emotional and cultural resources, and reduce them to sex objects*” (p. 226). In this psychological and cultural context, it is thus predictable that women would indicate more distress than men, and that this would be the case in general self-consciousness but in particular in sexual/body self-consciousness. There were statistically significant differences between the clinical and community samples. However, the size of these effects was small, and the clinical significance doubtful. It is perhaps more interesting to observe the degree of overlap between the clinical sample—receiving treatment for appearance altering conditions—and the community, who are not. Further work can determine whether this represents an unmet need in the community sample, or whether this group are addressing their appearance distress in alternative, non-medical ways.

Further analysis of the factor scores indicated that this two factor solution correlated with dominant area of appearance self-consciousness. There was a greater likelihood of significance and large effect sizes in SBSC for people who identified their main area of sensitivity in a region of their body that was sexually significant or concealable by clothing. There is clear evidence that increased body self-consciousness is related to decreased sexual satisfaction in the general population (Claudat & Warren, 2014). Issues concerning appearance and sexual difference for people with a visibly different appearance are also recognised as neglected areas such as in burns rehabilitation (Ahmad et al., 2013). Furthermore, there is evidence that in appearance altering conditions such as breast cancer (Taylor et al., 2013), professionals may not routinely attend to issues of sexuality, despite the reported willingness of patients to discuss it. Including an assessment such as the SBSC factor of DAS24 will facilitate these discussions and bring to the foreground for healthcare providers the potential impact of appearance on sexuality. The lack of understanding of sexual functioning in relation to body image, and any accompanying lack of measurement tools have been cited as a major barrier to developing effective interventions (Corry, Pruzinsky & Rumsey, 2009; Taylor et al., 2011).

Increased understanding of the factor analytic structure of DAS24 and the identification of a brief, six item subscale to measure SBSC adds to the tools available for research and intervention. The specificity of the SBSC factor was demonstrated by the differentiation of the sample according to sexually significant areas, while no difference was observed in SBSC in those concerned/unconcerned about non-sexually significant areas. It is beyond the scope of this paper to outline specific intervention strategies to work psychologically with those with higher scores on SBSC. Rather, we would advocate that those working therapeutically with clinical or general population groups are alert to differential levels of general versus sexual/body self-consciousness, and that visible differences around sexually significant areas of the body are potentially indicators of this. Furthermore, we would advocate a proportionate, needs-based intervention strategy based upon the graded model (Jenkinson, 2012) which incorporates a range of appearance related interventions from sociological and public health based through to prolonged individual complex case work. As such, the nature and timings of specific interventions based upon an identification of sexual/bodily self-consciousness will be diverse.

While a number of physical and psychological explanations have been put forward to explain individual differences in adjustment to visible differences (Moss & Rosser, 2012), as yet individual differences in relation to the way appearance impacts upon sexual functioning and sexual self-consciousness remains under-theorised. It is likely that models predicting body image satisfaction in the general (non-visibly different) population will be applicable—including the extent of internalisation of sexualised social ideals (e.g., McKenney & Bigler, 2014). Of course, other avenues could be explored to this end; initially, we would propose using the SBSC factor scores from the DAS24 to identify those at either end of a distribution of sexual and bodily self-consciousness, and purposively sample these to explore differences in sexualised self-concept.

A major strength of this study was its robustness, in terms of good data, a well-powered sample and close consideration of the most appropriate method of factor analysis. This permitted clear factor structure to emerge. Further validation of factor stability in other large samples, including a demonstration of a replication of the factor structure using CFA on a new sample would be useful. The extent to which the factors which emerged in this analysis are sensitive to any natural longitudinal change in self-consciousness, or sexuality, remains to be evaluated, and as such is a limitation of the current study. Furthermore, the extent to which under-reporting of sexual self-consciousness artificially deflates scores on the SBSC scale, due to a reticence to declare difficulties in such an emotive and sensitive area, remains to be further investigated.

## Conclusion

This brief paper adds further utility to a well-established measure, offering the possibility of using the complete scale, or one of the two subscales as required. It demonstrates the existence of a factor structure beyond the simple total score of the DAS24. The SBSC factor demonstrated greater sensitivity and specificity to distress which is based on a concern arising from sexually significant body areas.

## Supplemental Information

10.7717/peerj.1070/supp-1 Data S1 Key to data

10.7717/peerj.1070/supp-2 Data S2 Data—comma separated values
