# Supplementary material for: Identification of the underlying factor structure of the Derriford Appearance Scale 24
Source: PeerJ. 2015 Jul 2;3:e1070. doi: 10.7717/peerj.1070 (PMC4493685; doi:10.7717/peerj.1070)
Supplement: Data S1 [file peerj-03-1070-s001.docx]

Key:

| Column | Content |
| --- | --- |
| A | Age in years |
| B | Participant gender (1=female, 2=male, 3=not known)) |
| C-Z | DAS24 items (reverse scored) |
